# Ataxia telangiectasia: a review

**DOI:** 10.1186/s13023-016-0543-7

**Published:** 2016-11-25

**Authors:** Cynthia Rothblum-Oviatt, Jennifer Wright, Maureen A. Lefton-Greif, Sharon A. McGrath-Morrow, Thomas O. Crawford, Howard M. Lederman

**Affiliations:** 1 A-T Children’s Project, Coconut Creek, Florida USA; 2 The Ataxia Telangiectasia Clinical Center, Johns Hopkins Medical Institutions, Baltimore, Maryland USA; 3 The Ataxia Telangiectasia Clinical Center, Departments of Pediatrics and Pediatric Respiratory Sciences, Johns Hopkins Medical Institutions, Baltimore, Maryland USA; 4 The Ataxia Telangiectasia Clinical Center, Departments of Pediatrics and Neurology, Johns Hopkins Medical Institutions, Baltimore, Maryland USA; 5 The Ataxia Telangiectasia Clinical Center, Departments of Pediatrics, Medicine and Pathology, Johns Hopkins Medical Institutions, Baltimore, Maryland USA

**Keywords:** Cancer, Neurodegeneration, Cerebellum, Purkinje cells, Immunodeficiency, Dysphagia, Pulmonary disease

## Abstract

**Definition of the disease:**

Ataxia telangiectasia (A-T) is an autosomal recessive disorder primarily characterized by cerebellar degeneration, telangiectasia, immunodeficiency, cancer susceptibility and radiation sensitivity. A-T is often referred to as a genome instability or DNA damage response syndrome.

**Epidemiology:**

The world-wide prevalence of A-T is estimated to be between 1 in 40,000 and 1 in 100,000 live births.

**Clinical description:**

A-T is a complex disorder with substantial variability in the severity of features between affected individuals, and at different ages. Neurological symptoms most often first appear in early childhood when children begin to sit or walk. They have immunological abnormalities including immunoglobulin and antibody deficiencies and lymphopenia. People with A-T have an increased predisposition for cancers, particularly of lymphoid origin. Pulmonary disease and problems with feeding, swallowing and nutrition are common, and there also may be dermatological and endocrine manifestations.

**Etiology:**

A-T is caused by mutations in the *ATM* (Ataxia Telangiectasia, Mutated) gene which encodes a protein of the same name. The primary role of the ATM protein is coordination of cellular signaling pathways in response to DNA double strand breaks, oxidative stress and other genotoxic stress.

**Diagnosis:**

The diagnosis of A-T is usually suspected by the combination of neurologic clinical features (ataxia, abnormal control of eye movement, and postural instability) with one or more of the following which may vary in their appearance: telangiectasia, frequent sinopulmonary infections and specific laboratory abnormalities (e.g. IgA deficiency, lymphopenia especially affecting T lymphocytes and increased alpha-fetoprotein levels). Because certain neurological features may arise later, a diagnosis of A-T should be carefully considered for any ataxic child with an otherwise elusive diagnosis. A diagnosis of A-T can be confirmed by the finding of an absence or deficiency of the ATM protein or its kinase activity in cultured cell lines, and/or identification of the pathological mutations in the *ATM* gene.

**Differential diagnosis:**

There are several other neurologic and rare disorders that physicians must consider when diagnosing A-T and that can be confused with A-T. Differentiation of these various disorders is often possible with clinical features and selected laboratory tests, including gene sequencing.

**Antenatal diagnosis:**

Antenatal diagnosis can be performed if the pathological *ATM* mutations in that family have been identified in an affected child. In the absence of identifying mutations, antenatal diagnosis can be made by haplotype analysis if an unambiguous diagnosis of the affected child has been made through clinical and laboratory findings and/or ATM protein analysis.

**Genetic counseling:**

Genetic counseling can help family members of a patient with A-T understand when genetic testing for A-T is feasible, and how the test results should be interpreted.

**Management and prognosis:**

Treatment of the neurologic problems associated with A-T is symptomatic and supportive, as there are no treatments known to slow or stop the neurodegeneration. However, other manifestations of A-T, e.g. immunodeficiency, pulmonary disease, failure to thrive and diabetes can be treated effectively.

## Background

Ataxia telangiectasia, or A-T, is also referred to as Louis-Bar Syndrome (OMIM #208900). Orphanet Orpha Number: ORPHA100. A-T was given its commonly used name by Elena Boder and Robert P. Sedgwick, who in 1957 described a familial syndrome of progressive cerebellar ataxia, oculocutaneous telangiectasia and frequent pulmonary infection [1].

### Definition

A-T is an autosomal recessive cerebellar ataxia [2]. It has also been widely referred to as a genome instability syndrome, a chromosomal instability syndrome, a DNA repair disorder, a DNA damage response (DDR) syndrome and, less commonly, as a neurocutaneous syndrome. A-T is characterized by progressive cerebellar degeneration, telangiectasia, immunodeficiency, recurrent sinopulmonary infections, radiation sensitivity, premature aging, and a predisposition to cancer development, especially of lymphoid origin. Other abnormalities include poor growth, gonadal atrophy, delayed pubertal development and insulin resistant diabetes [3]. It is important to note that A-T is a complex disease and not all people have the same clinical presentation, constellation of symptoms and/or laboratory findings (e.g. telangiectasia are not present in all individuals with A-T, see Clinical Description below) [4].

Cells derived from patients with A-T demonstrate sensitivity to ionizing irradiation, chromosomal instability, shortened telomeres, premature senescence and a defective response to DNA double strand breaks (DSBs) (reviewed in [5] and [6] and more recently in [7]).

### Epidemiology

With the exception of consanguineous populations, individuals of all races and ethnicities are affected equally by A-T. The prevalence is estimated to be <1–9/100,000, although incidences as high as 1 in 40,000 [8, 9] and as low as approximately 1 in 300,000 [9] have been reported.

### Clinical description

Because not all children develop in the same manner or at the same rate, the diagnosis of A-T may not be made until the early school years when the neurologic symptoms (impaired gait, hand incoordination, abnormal eye movements), and the telangiectasia appear or become worse. Different forms or presentations of A-T have been described in the literature, with those more severe variably categorized as “classic”, “typical”, “early onset” or “childhood onset” A-T, while milder forms have been referred to as “variant”, “atypical”, “late onset” or “adult onset” A-T. We utilize the terms “classic” and “mild” to distinguish the two different, but broadly recognized, clinical presentations of A-T. People with mild A-T present with less severe, later onset manifestations associated with longer survival (for a more detailed comparison of the classic and mild clinical forms of A-T see Etiology: Genotype/Phenotype Correlations).

#### Ataxia and other neurological manifestations

In the classic presentation of the disease, ataxia first appears during the toddler stage when children begin to sit and walk. Children with A-T often start walking at a normal age but then fail to improve much from their initial wobbly gait (reviewed in [4, 10] and [11]). Often they have problems standing or sitting still and tend to sway slowly from side to side or backwards. Because most children with classic A-T have stable neurologic symptoms for the first 4–5 years of life, they initially may be labeled as having “ataxic cerebral palsy” [10], but the existence of such a syndrome is nosologically unclear.

In primary school years, walking becomes more difficult, and children will use doorways and walls for support. Children with A-T often run or walk quickly, and do so with a curiously narrow stance, in deference to walking more carefully and slowly. Around the beginning of their second decade children with classic A-T start using a wheelchair in the community setting [4]. During school years children often have increasing difficulty with reading because of impaired coordination of eye movement (see “Eye and Vision” below). At the same time, other problems with fine motor functions (writing, coloring, and using utensils to eat) and with dysarthria may arise ([4, 12]). Drooling may persist beyond expected ages and particularly in young children when they are tired or concentrating. Most of these neurologic problems stop progressing after the age of about 12–15 years.

At any age, however, individuals with A-T may develop increasing difficulty with involuntary movements. These can take many forms, including chorea, athetosis, dystonia, myoclonic jerks, or various tremors including rhythmic and non-rhythmic movements that complicate intended movements [13, 14]. Other extrapyramidal symptoms may include body hypokinesia or bradykinesia and facial hypomimea ([4, 11]).

Distal to proximal advancing loss of tendon reflexes is also characteristic of A-T [8], reflecting a progressive sensory and motor neuropathy [4, 15].

**Fig. 1:**
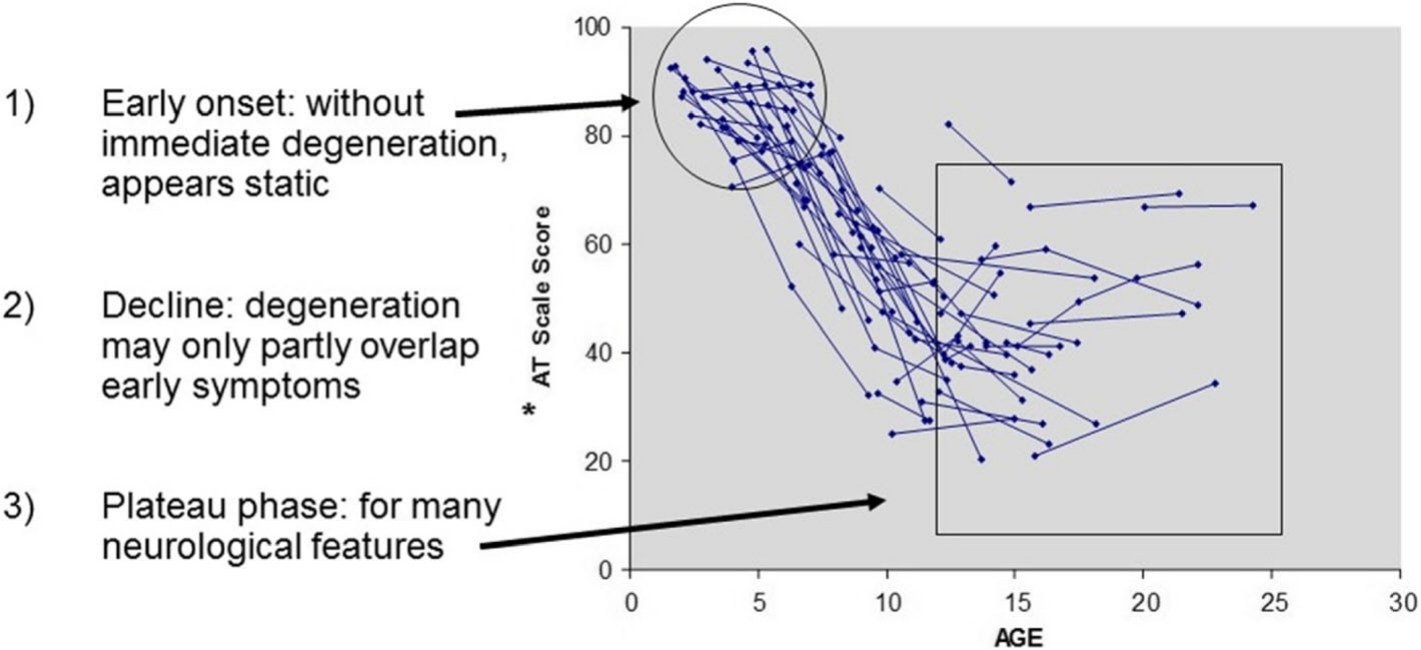
The Pattern of Neurologic Decline in Classic A-T [173]. * AT Scale Scores based on the Crawford Quantitative Neurologic A-T Scale [174]; 100 = Normal

Although many systemic complications can create a complex clinical picture, the distinct pattern of neurological decline associated with the classic presentation of A-T is depicted in Fig. 1.

#### Neuroimaging findings

The neuropathological hallmark of A-T is diffuse degeneration or atrophy of the cerebellar vermis and hemispheres, involving Purkinje cells (PCs) and, to a lesser extent, granule neurons. Various neuropathological abnormalities (e.g. neuronal changes, gliosis and vascular changes) have also been observed in the cerebrum, brain stem and spinal cord. (reviewed extensively in [10] and more recently in [16]).

Although early neuroimaging studies in A-T were performed using computed tomography (CT), for technical reasons, and because of the requirement for radiation, magnetic resonance imaging (MRI) is the preferred modality for visualizing the central nervous system (CNS) and spinal cord in A-T. Studies utilizing T1- and T2-weighted MRI and more recently diffusion MRI (dMRI) have been published [16].

For the majority of people with A-T, neuroimaging studies in the toddler years and early child years are normal ([11] and unpublished observations). As the disease progresses, MRI studies support the pathological finding of variable, progressive and diffuse cerebellar atrophy [16]. Between patients it is notable that the magnitude of volume loss correlates poorly with clinical features (unpublished observations).

In addition to cerebellar atrophy, MRI studies have demonstrated cerebral, white matter abnormalities in older patients, including hemosiderin deposits and deep cerebral telangiectatic vessels, as well as degenerative changes in white matter corticomotor tracts extending from the cerebellum in younger patients with A-T [17–19].

Magnetic resonance spectroscopy (MRS) studies to measure the levels of various brain metabolites have also been performed for A-T, although with somewhat conflicting results [20, 21]. Lin et al. found decreased levels of all analyzed metabolites (N-acetyl aspartate [NAA], choline [Cho], and creatine [Cr]) in the cerebellar vermis with a trend towards decreased metabolite levels in the cerebellar hemispheres [20], whereas Wallis et al. observed increased levels of Cho in the cerebellum of adults with A-T [21].

A positron emission tomography (PET) study to measure brain glucose metabolism in individuals with A-T has also been performed [22]. Due to the radiation exposure inherent in PET imaging, participants in this study were restricted to 18 years of age or older. Although glucose metabolism was uniformly reduced in the cerebellum of patients with A-T, increased metabolism observed in the globus pallidus was associated with decreased motor performance. Additional imaging studies are warranted; however, these results suggest that deep brain stimulation (DBS) targeting the pallidus may be a therapeutic option for A-T [22].

#### Telangiectasia

**Fig. 2:**
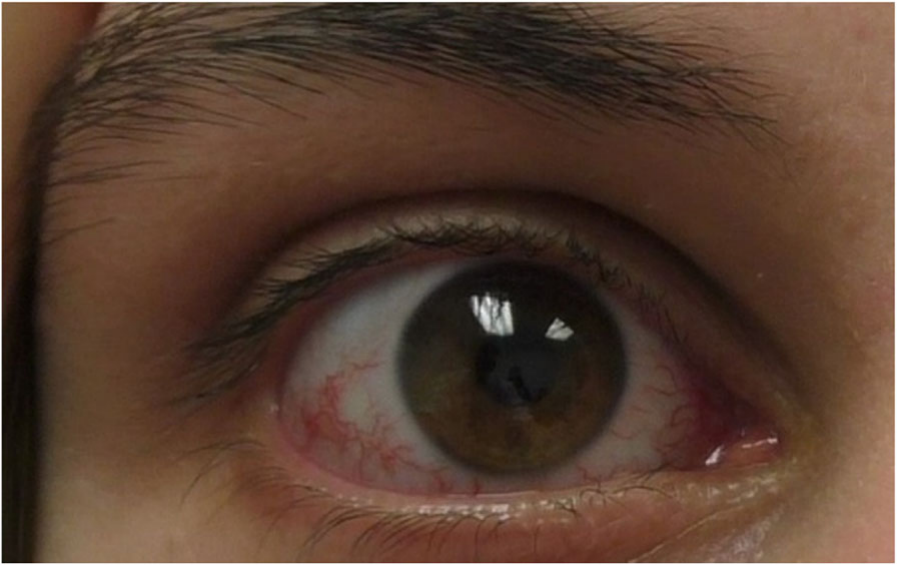
Ocular Telangiectasia in a Person with A-T

Telangiectasia within the bulbar conjunctiva over the exposed sclera of the eyes usually occur by the age of 5–8 years, but sometimes later or not at all (Fig. 2) [23]. The absence of telangiectasia does not exclude the diagnosis of A-T. Although potentially a cosmetic problem, the ocular telangiectasia do not bleed or itch though they are sometimes misdiagnosed due to chronic conjunctivitis or allergy. It is their constant nature, not changing with time, weather or emotion, which marks them as different from other visible blood vessels. Telangiectasia can also appear on sun-exposed areas of skin, especially the face and ears. They occur in the bladder as a late complication of chemotherapy with cyclophosphamide, have been seen deep inside the brain of older people with A-T [17], and occasionally arise in the liver and lungs (unpublished observations).

#### Eye and vision

The telangiectasia do not effect vision and visual acuity is normal in A-T [24]. However, control of eye movement and visual fixation is often impaired affecting functions that require fast, accurate eye movements from point to point (e.g. reading). Abnormal eye movements associated with A-T include: oculomotor apraxia, nystagmus (including horizontal nystagmus in primary gaze, nystagmus on lateral gaze, post-rotary nystagmus and periodic alternating nystagmus), hypometric saccades and saccadic intrusions, convergence/accommodation and VOR abnormalities [25–27]. Strabismus is common. There may be difficulty in coordinating eye position and shaping the lens to see objects clearly at close distances.

#### Immunological manifestations

About two-thirds of people with A-T have abnormalities of the immune system [28, 29]. The most common abnormalities are low levels of one or more classes of immunoglobulin (IgG, IgA, IgM or IgG subclasses), failure to make antibodies in response to vaccines or infections, and lymphopenia, especially affecting T-lymphocytes. There are reduced numbers of new B cells leaving the bone marrow and new T cells leaving the thymus [30], reduced proportions of naive B and T cells, and reduced antigen receptor repertoire [29]. A small percentage of people with A-T also may have elevated levels of IgM in combination with IgG and/or IgA deficiency. When this is the presenting symptom in infant- or childhood, the diagnosis of A-T can be confused with that of hyper-IgM syndrome [31]. In the majority of individuals with A-T, the immunologic abnormalities do not deteriorate over time, but approximately 10% will develop more severe problems most often with humoral immunity [28, 32].

Sinopulmonary infections are common in people with A-T [28, 33, 34]. All children with A-T should have their immune systems evaluated to detect those with severe problems that require treatment to minimize the number or severity of infections.

People with A-T have an increased risk of developing autoimmune or chronic inflammatory diseases. This risk is probably a secondary effect of their immunodeficiency and not a direct effect of the lack of ATM protein. The most common examples of such disorders in A-T include immune thrombocytopenia (ITP), several forms of arthritis, and vitiligo.

Fewer than 10% of people with A-T develop chronic cutaneous granulomas that are thought to be due to disordered inflammation [35, 36].

#### Pulmonary manifestations

Chronic lung disease develops in more than 25% of people with A-T [37]. Lingering cough, chest congestion and/or wheeze may be early symptoms of underlying lung disease in a person with A-T. These symptoms may occur in the absence of other systemic symptoms, resulting in delayed treatment. If respiratory symptoms are ignored, severe manifestations of lung disease can occur which include bronchiectasis, recurrent pneumonia, lung fibrosis and interstitial lung disease (ILD). Although not always avoidable, some of these conditions can be prevented by recognition of cause and early treatment [38].

Immune dysregulation in A-T can lead to recurrent pneumonia, bronchiectasis and ILD. Poor mucociliary clearance from an inadequate cough and chronic aspiration from impaired bulbar function can increase severity of chronic respiratory symptoms.

Restrictive lung disease is common in A-T and is characterized by lower than normal forced vital capacity (FVC) [39, 40]. Low FVC and decreased pulmonary reserve in people with A-T can increase their risk for pulmonary complications from respiratory illnesses, systemic stress and anesthetic procedures for surgery. Identifying those with restrictive lung disease can help providers avoid respiratory complications during elective and non-elective anesthesia and surgery [41]. Causes of restrictive lung disease in A-T include respiratory muscle weakness, impaired coordination of muscles involved in respiration and ILD [42]. Shortened telomeres and sensitivity to ionizing radiation are also characteristic of A-T and can increase the risk of complications such as pulmonary fibrosis when treating malignancies [43, 44].

Two studies have found an association between higher systemic levels of the pro-inflammatory cytokines IL6 and IL8 and lower percent FVC in people with A-T suggesting a link between inflammation and lung decline in this disease [45, 46].

#### Cancer

People with A-T have a highly increased incidence (approximately 25% lifetime risk [47, 48]) of cancers. Lymphomas and leukemias most often occur in people with classic A-T less than 20 years of age, but adults are susceptible to both lymphoid tumors and a variety of solid tumors including breast, liver, gastric and esophageal carcinomas (unpublished observations). An extensive analysis of the types of cancers that occur in both the classic and mild forms of the disease has been performed on combined cohorts from the UK and the Netherlands [47].

There is as yet no way to predict which individuals with A-T will develop cancer, and unlike surveillance for many solid tumors (e.g. mammography, colonoscopy, PSA levels), there are no accepted methods to provide surveillance for lymphomas and leukemias. Hematopoietic cancer must be considered as a diagnostic possibility whenever potential symptoms (e.g. persistent swollen lymph nodes, unexplained fever) arise.

#### Cancer in A-T carriers

Carriers, those who have one mutated copy of the *ATM* gene, such as the parents of a person with A-T, are generally healthy. However, a systematic meta-analysis found that *ATM* mutation carriers have a reduced lifespan due to cancer (breast and gastrointestinal tract) and ischemic heart disease [49].

In particular, *ATM* is considered a moderate risk or moderate penetrance breast cancer susceptibility gene [50, 51]. Female carriers are considered to have an approximately 2.3 fold increased risk for the development of breast cancer compared to the general population [51–53]. A 2016 meta-analysis found the cumulative risk of breast cancer in carriers to be approximately 6% by age 50 and approximately 30% by age 80 [54]. Standard breast cancer surveillance, including monthly breast self-exams and mammography at the usual schedule for age, is recommended unless an individual has other risk factors (e.g., family history of breast cancer).

#### Radiation sensitivity

People with A-T have an increased sensitivity to ionizing radiation (X-rays and gamma rays), which can be cytotoxic. X-ray exposure should be limited to times when it is medically necessary for diagnostic purposes. Radiation therapy for cancer or any other reason is generally harmful for individuals with A-T and should be performed only in rare circumstances and at reduced doses [55, 56]. Although A-T cells in culture have an altered DNA damage response to other genotoxic agents (e.g. ultraviolet [UV] light) [57, 58], individuals with A-T do not have an increased incidence of skin cancer and can cope normally with sun exposure, so there is no need for special precautions for exposure to sunlight.

#### Radiation sensitivity in carriers

Cultured cells from heterozygote carriers of *ATM* mutations have been reported to have a variable but “intermediate” sensitivity to radiation, being more sensitive than normal control cells but less sensitive than homozygous ATM null cells [59–61]. Clinically, a 1998 study of heterozygotes in families with A-T demonstrated no hypersensitivity to therapeutic radiation for carriers with prostate and breast cancer [62]. Although one study reported that women who possess specific rare pathological *ATM* missense variants and who receive therapeutic radiation may have an elevated risk for developing contralateral breast cancer [63], this precaution will not apply to the majority of carriers who develop breast or any other cancer. In our opinion, cancer therapy in A-T carriers should be based on what is considered the current and best curative option.

#### Feeding, swallowing, and nutrition

Feeding and swallowing (deglutition) may become difficult for people with A-T as they age [64]. Primary goals for feeding and swallowing are safe, adequate, and enjoyable mealtimes. Involuntary movements can make self-feeding difficult and result in messy or excessively prolonged mealtimes. In general, meals longer than 30 min may be stressful, interfere with other daily activities, and compromise hydration and nutritional intake.

Dysphagia is common in A-T and typically appears during the second decade of life because of the neurological changes which interfere with the coordination of mouth and pharynx movements necessary for safe and efficient swallowing [64]. Coordination problems involving the mouth may make chewing difficult and increase the duration of meals. Problems involving the pharynx may cause aspiration of liquid, food, and saliva. Dysphagia with concomitant silent aspiration may cause lung problems because of impaired clearance of food or liquids from the airway.

Dysphagia also can result in nutritional compromise because the process of eating becomes slow and difficult. Some people with A-T stop eating or reduce their intake at meals because of frustration or fatigue with the process. Insufficient caloric intake may compromise growth in children and weight maintenance in older persons, contributing to lower body mass indices (BMI) in comparison to healthy, age-matched individuals [65–69]. Poor nutrition may exaggerate the presentation of neurologic disability. Abnormal respiratory-swallowing coupling has been associated with an increased risk for aspiration and may signify swallowing problems prior to the development of nutritional and pulmonary sequelae in A-T [70]. Warning signs of a problem with deglutition are presented in Table 1.

**Table 1:** Warning Signs of a Swallowing Problem in A-T

• Choking or coughing when eating or drinking	
• Poor weight gain during ages of expected growth or weight loss at any age	
• Excessive drooling	
• Mealtimes longer than 40–45 min, on a regular basis	
• Foods or drinks previously enjoyed are now refused or difficult	
• Chewing problems	
• Increase in the frequency or duration of breathing or respiratory problems	
• Increase in lung infections	

#### Endocrine abnormalities

##### Poor growth

Poor growth is a common feature of A-T. Nutritional compromise, infections and altered growth factor and hormone levels have been proposed to contribute to this growth impairment [71, 72]. A study of endocrine abnormalities in an Israeli cohort of patients with A-T demonstrated that growth impairment was present in infancy, prior to the onset of neurological symptoms and the nutritional problems commonly seen as children age. This study also showed that impaired growth was more prominent in females than males, and that this difference is apparent at an age before gonadotropins begin to affect growth rates [3].

##### Delayed pubertal development/gonadal dysgenesis

Infertility is often described as a facet of A-T. Whereas this is certainly the case for the mouse models of A-T [73–76], in humans it may be more accurate to describe the reproductive abnormalities as gonadal atrophy or dysgenesis causing delayed pubertal development and early menopause. Abnormalities in gonadal development and function appear to be more prominent in females than males [3]. We are aware of pregnancies in people with mild forms of A-T ([77] and unpublished observations), but not in anyone with the classic form of the disease.

##### Insulin-resistant diabetes

A minority of patients with A-T suffer from insulin resistant diabetes which typically appears as a late event during disease progression. Of note, reduced insulin sensitivity and dysglycemia may be observed in individuals with A-T who do not have diabetes [78].

#### Hair and skin

A-T can cause features of early aging such as premature graying of the hair [4]. People with A-T can also have an increased prevalence of vitiligo, and warts that can be extensive and recalcitrant to treatment ([28] and unpublished observations).

#### Sleep

Interestingly, unlike other neuromotor disorders, such as Duchenne Muscular Dystrophy, overnight polysomnography has not identified regular sleep-related gas exchange abnormalities in patients with A-T. The majority of subjects studied were noted to have decreased sleep efficiency which has been associated with chronic disease states [79].

#### Cognition

Very few neuropsychological studies have been performed in individuals with A-T. One study performed in 2000 demonstrated deficits in the judgement of duration (i.e. the “judgement of explicit time intervals” or perceptual timing) [80].

Subsequent studies demonstrated that certain cognitive deficits appear relatively early in A-T, then become broader and more profound during later stages of the disease [81, 82]. In these studies specific impairments were observed in intellectual functioning, nonverbal memory, verbal abstract reasoning and calculation, and executive function. Pronounced deficits in perceptual timing were also observed; however language functioning was not impaired and “expressive language” was noted as a strength in children with A-T, even during later stages of the disease. The cognitive impairments seen in A-T have been found to be characteristic of Cerebellar Cognitive Affective Syndrome (CCAS) [83, 84].

#### Orthopedic manifestations

Acquired deformity of the feet is common in people with A-T (unpublished observations) and compounds the difficulty individuals have with walking due to impaired coordination. Scoliosis also occurs ([85] and unpublished observations), but is relatively uncommon. Occasionally, individuals with A-T develop contractures of the fingers, most often because of inflammatory connective tissue disease, but sometimes from neuropathy.

#### Manifestations in aging or older patients with A-T

Certain problems occur with an unexpectedly high frequency in patients with A-T who survive into their twenties and beyond. Table 2 provides a list of these types of problems.

**Table 2:** Problems Observed in Aging or Older People with A-T

• Ballistic, retropulsive or jerky movements	
• Sensory and motor neuropathy	
• Brain telangiectasia (observed by MRI)	
• Restrictive lung disease	
• Elevated cholesterol and triglyceride levels	
• Glucose intolerance and diabetes	
• Liver abnormalities (e.g. fatty liver; non-alcoholic cirrhosis; elevated serum transaminases)	
• Changes in the types of malignancies (there is an increased incidence for both lymphoid and solid tumors)	
• Osteoporosis/osteopenia and low vitamin D levels	
• Postural scoliosis and progressive foot deformities	
• Gastroesophageal reflux (especially if reflux was an issue in infanthood)	
• Early menopause	
• Depression	
• Aging parents and caregivers	

Of particular note, liver abnormalities, such as elevated serum transaminase levels, steatosis and non-alcoholic cirrhosis including fibrotic changes have been observed as people with A-T age, as have elevated triglyceride and cholesterol levels [3, 86, 87].

The spectrum of malignant disease is also different in older individuals with classic A-T, as there is an increased risk for the development of both lymphoid malignancies and solid tumors in people over the age of 20 (unpublished observations).

#### Other manifestations of A-T

Some people with A-T suffer from bladder and/or bowel incontinence that results from difficulties with transfers rather than a length-dependent neuropathy. Some individuals also go through a period of recurrent vomiting which appears to be more prevalent in the mornings. This transient but repeated vomiting may correlate with the development of eye movement abnormalities, as people can have a sensation of motion sickness or dizziness with head movement. This symptom can be treated with drugs for motion sickness and usually resolves in a period of months, possibly as the eye movement abnormalities become more severe. ([88] and unpublished observations).

### Etiology

#### Genetics

The mode of inheritance for A-T is autosomal recessive. A-T is caused by mutations in the *ATM* (ataxia telangiectasia, mutated) gene which was cloned by Savitsky et al. in 1995 [89]. *ATM* is located on human chromosome 11q22-q23 [90] and is made up of 66 exons (four non-coding and 62 coding) spanning 150 kb of genomic DNA.

#### Genotype / phenotype correlations

The *ATM* gene is large, and although certain populations contain a higher frequency of identical mutations due to the founder effect [91, 92], there is no one area of the gene especially susceptible to mutation. Mutations have been identified in the proximal, central and distal regions of the human *ATM* gene [92]. These include primarily nonsense mutations and frame shifts resulting from insertions and deletions, but also missense and leaky splice-site mutations. Compound heterozygosity is common [93].

In 1998, a genotype/phenotype analysis was performed on a small cohort of individuals who had less severe clinical presentations of A-T [94]. Subsequently, other analyses of genotype/phenotype correlations in disease severity and in the development of cancer were performed on larger A-T cohorts ([47, 95, 96] and reviewed in [97]).

Briefly, the majority of *ATM* mutations are truncating [98, 99], creating highly unstable protein fragments. In such cases, ATM protein cannot be detected by western blotting and ATM kinase activity is not observed. Individuals who possess these mutations have a classic clinical presentation of A-T, and the severity of their disease follows a relatively predictable course (see Fig. 1 and Table 3). Individuals with A-T possessing residual ATM protein (observable by western blot) that lacks kinase activity also may present with this classic phenotype [95].

**Table 3:** Classic vs. Mild Forms of A-T

	Classic Form	Mild Form
Neurological Manifestations	Neurological deficits are typically observed during the toddler years resulting in wheelchair dependency around the age of 10.	Individuals have more mild neurological deficits in childhood with slower age-related neurodegeneration. The predominant neurological symptoms or symptoms to present first may be myoclonus, dystonia, choreoathetosis or tremor with ataxia appearing later [175–177]. Oculomotor apraxia may also appear later or not at all [95].
Immunodeficiencies	Roughly two-thirds of people with classic A-T suffer from some type of immunodeficiency and/or lymphopenia.	Immunodeficiencies do occur, but are less common.
Pulmonary Disease	Relatively common.	Less common.
Cancer	Although malignancies in these individuals tend to occur at a younger age and are often lymphoid in nature, cancers in older individuals do occur and include both hematopoietic and non-hematopoietic malignancies.	Malignancies tend to appear later in life and include a higher proportion of non-hematopoietic cancers. The diagnosis of cancer can precede the diagnosis of A-T.

Certain missense mutations, in-frame mutations or leaky splice-site mutations allow for the production of residual amounts of functioning ATM protein [97]. ATM protein can be detected on western blots and some level of kinase activity is present. Individuals who possess these types of *ATM* mutations have traditionally been referred to as “atypical” or “variant,” and more recently as “mild.” Because there is some degree of residual ATM function, from either normal or mutant protein, the overall severity of their clinical course is less, and the progression of their disease is slower (Table 3). Of note, in the mild form of the disease, the diagnosis of cancer can precede the diagnosis of A-T [100, 101]. As radiation therapy and radiomimetic chemotherapy can be especially cytotoxic in individuals with this disease, a diagnosis of A-T should be considered for any individual with cancer who has an undiagnosed disorder associated with gait disturbance or eye movement abnormality, especially if the symptoms are progressive.

Mild cases also have been reported to present with neurological symptoms in adulthood versus childhood [95, 102–105]; however, in at least one case report the authors could not definitively rule out the possibility that mild neurological abnormalities existed in childhood [102].

Interestingly, three documented “null” milds have been reported in the literature [77, 106]. The neurological presentation and progression of their disease is mild. However, these patients have null *ATM* mutations (frameshift and splice site mutations causing truncation), no ATM protein detectable by western blot analysis, no kinase activity and the typical cellular phenotype for classic A-T. Therefore, these individuals somehow compensate for the absence of functioning ATM protein. Although rare, these patients are of particular interest because the genetic and/or environmental factors that modify the severity of their clinical course may represent targets for treatment interventions.

Other A-T “variants” were described earlier in 1992 [107]. These individuals possessed a classic clinical presentation but an intermediate cellular radiosensitivity phenotype. Given that some individuals, albeit rare, can present with a mild disease course but classic cellular radiosensitivity, it appears that clinical severity does not always correlate with the in vitro radiation sensitivity of cultured cells.

#### Pathophysiology: how does loss of the ATM protein create a multisystem disorder?

The *ATM* gene encodes a large 3056 amino acid protein of the same name whose best known, and arguably most well understood, role is coordinating the cellular response to DNA DSBs. However, the ATM kinase also responds to oxidative stress, other forms of genotoxic stress and other stressors that affect cellular homeostasis, resulting in the direct phosphorylation and regulation of an ever-growing list of downstream substrates ([108, 109] and reviewed in [110]). A summary of the features of the ATM protein is presented in Table 4.

**Table 4:** The ATM Protein (reviewed in [110])

• 3056 amino acids	
• Serine/Threonine protein kinase	
• Member of the family of PI3 Kinase-like Kinases (PIKKs)	
• Located primarily in the nucleus; smaller amounts in the cytoplasm and associated with mitochondria and peroxisomes [178]	
• Activated primarily by DSBs and oxidative stress, but also agents affecting chromatin organization, hypoxia, hypotonic stress and hyperthermia	
• Phosphorylates and regulates a variety of protein substrates involved in o The DNA damage response (NHEJ and HRR) to DSBs o Various other genotoxic stress responses o DNA repair processes o Cell cycle checkpoints o Other cell stress responses o Apoptosis	

*NHEJ* non-homologous end-joining, *HRR* homologous recombination repair

##### Cancer

In the absence of the ATM protein, the signaling network that responds to DNA DSBs is defective, and responses to other types of genotoxic stress are reduced to various degrees. The result is genomic instability which can lead to the development of cancers [6].

##### Radiosensitivity

Irradiation (e.g. radiation therapy for cancers) and radiomimetic compounds (e.g. those used in cancer chemotherapy protocols) induce DSBs and other DNA lesions whose repair is severely impaired when ATM is absent. Consequently, such agents can prove especially cytotoxic to people with A-T.

##### Immune system defects and immune-related cancers

As lymphocytes develop they undergo gene rearrangements to generate clonal diversity and class switch recombination, processes which generate DSBs. In the absence of ATM, the effective repair of these DSBs is difficult [111–113]. As a result many people with A-T have reduced numbers of lymphocytes and some impairment of lymphocyte function (such as an impaired ability to make antibodies in response to vaccines or infections) [28, 29]. In addition, chromosomal translocations can occur as a result of aberrant DSB repair, making these cells prone to the development of cancer (lymphomas and leukemias) [114, 115] (see Table 5).

**Table 5:** ATM and the Immune System

ATM is involved in: o V(D)J recombination in the production of immunoglobulins and α/β chain recombination in the production of T cell receptors [111, 112] o Class switch recombination in B cells [179, 180] o T cell proliferation and survival following T cell receptor stimulation [181, 182]	
ATM deficiency results in: o Low immunoglobulin levels (particularly IgA, IgG subclasses and IgE) o Lymphopenia (particularly affecting T cell numbers) o Decreased immune repertoire diversity o Genomic instability and translocations which can result in lymphoid malignancies	

Interestingly, treatment of Atm deficient mice with antioxidants such as tempol, N-acetyl cysteine (NAC) or the nitroxide antioxidant CTMIO delays the onset of thymic lymphoma [116–118], suggesting that oxidative stress characterized by elevated ROS and/or abnormal redox signaling plays some role in lymphomagenesis in these animals and perhaps humans.

##### Neurodegeneration

A-T is one of several DNA repair disorders which results in neurological abnormalities and/or neurodegeneration (reviewed in [119–121]). Arguably some of the most devastating symptoms of A-T are a result of progressive cerebellar degeneration, characterized by the gradual loss and/or aberrant location of PCs and, to a lesser extent, the gradual loss of granule cells [122, 123]. The cause of this cell death is not known, though many hypotheses have been proposed (reviewed in [11]). Current hypotheses to explain the neurodegeneration associated with A-T are summarized in Table 6. Much of the evidence in existence to date supports the idea that a defective response to genotoxic and/or oxidative stress contributes to the neuronal cell dysfunction and death in A-T. However, the hypotheses in Table 6 may not be mutually exclusive and more than one of these mechanisms may underlie neuronal cell death when there is an absence or deficiency of ATM.

**Table 6:** Hypotheses to Explain the Neurodegeneration in A-T

• Defective DDR [183, 184] or repair resulting in: o the failed clearance of genomically damaged neurons during development [76, 185] o transcription stress [119] and abortive transcription involving topoisomerase 1 cleavage complex (TOP1cc) dependent lesions [186–189] o aneuploidy [190]
• Defective response to oxidative stress characterized by elevated ROS and altered cellular redox status [191–194] and reviewed in [11, 195, 196]
• Mitochondrial dysfunction [197–199] and reviewed in [11]
• Defects in neuronal function involving: o Failed cell cycle regulation resulting in the re-entry of post-mitotic (mature) neurons into the cell cycle [200] o Synaptic/vesicular dysregulation [201–203] o Altered epigenetics including − HDAC4 nuclear translocation [204] − Histone H3 hypermethylation [205] and − Reduced 5-hydroxymethylcytosine [206]
• Defects in brain vasculature [207]
• Altered protein turnover [208]

*DDR* DNA damage response

Importantly, the loss of cerebellar cells does not explain all of the neurologic abnormalities seen in people with A-T, and the effects of ATM deficiency on the other areas of the brain outside of the cerebellum are being actively investigated.

##### Pulmonary disease

In addition to the neurological deficits which contribute to bulbar weakness and the immunodeficiencies which can contribute to susceptibility to chronic sinopulmonary infections, several other factors may influence the development of pulmonary disease in A-T. These include premature aging, inflammation, oxidative stress and an inability to properly repair damage that occurs in the lungs over time [124, 125]. Telomere shortening is also a feature of A-T and has been found to be associated with both idiopathic and genetically based ILDs [126].

##### Gonadal dysgenesis

Because programmed DSBs are generated to initiate meiosis, meiotic defects and arrest can occur when ATM is not present ([127] and reviewed in [5]) and may contribute to the gonadal dysgenesis associated with A-T.

##### Progeric changes

Cells from people with A-T demonstrate genomic instability, slow growth and premature senescence in culture, shortened telomeres and an on-going, low level genotoxic stress response [128–130]. These factors may contribute to the progeric changes of skin and hair sometimes observed in people with A-T. For example, DNA damage and genomic instability cause melanocyte stem cell (MSC) differentiation which produces graying. Thus, ATM may act as a “stemness checkpoint” protecting against MSC differentiation and premature graying of the hair [131]. An extensive review of this aspect of A-T, including the various biochemical pathways underlying it, has been performed [132].

##### Insulin-resistant diabetes

The finding that insulin signaling induces ATM-dependent phosphorylation of 4E-BP1 was published in 2000 [133]. Since that time, others have demonstrated that the insulin and insulin-like growth factor 1 (IGF-1) / IGF-1 receptor axes are affected by the loss of ATM in cell models, *Atm*−/− mice and in patients with A-T (recently reviewed in [134] and [135]). Further, the loss of Atm protein in ApoE−/− mice increases insulin resistance and exacerbates other features of metabolic syndrome [136]. Therefore, the role of ATM in insulin and IGF-1 metabolic signaling may explain the diabetic phenotype sometimes seen in A-T.

##### Increased alpha-fetoprotein (AFP) levels

AFP levels are very high in all newborns, and normally descend to adult levels over the first year to 18 months. Approximately 95% of people with A-T have elevated serum AFP levels after the age of two, and measured levels of AFP appear to increase slowly over time [137]. Why the majority of individuals with A-T have elevated levels of AFP remains unknown.

##### Appearance of telangiectasia

The cause of telangiectasia or dilated, enlarged blood vessels in the absence of the ATM protein is not yet known.

### Diagnosis

Because A-T is so rare, doctors may not be familiar with the symptoms or criteria for making a diagnosis. The late appearance of telangiectasia may also be a barrier to diagnosis.

A diagnosis of A-T can usually be made by the combination of clinical features and specific laboratory abnormalities. A variety of abnormal laboratory findings occur in most people with A-T, but not all abnormalities are seen in all patients. These abnormalities are listed in Table 7.

**Table 7:** Laboratory Abnormalities in A-T

• Elevated and slowly increasing serum alpha-fetoprotein levels after two years of age
• Low serum levels of immunoglobulins (IgA, IgG, IgG subclasses, IgE) and lymphopenia (particularly affecting T-lymphocytes)
• Spontaneous and X-ray induced chromosomal breaks and rearrangements in cultured lymphocytes and fibroblasts
• Reduced survival of cultured lymphocytes and fibroblasts after exposure to ionizing radiation [209]
• Cerebellar atrophy detected by MRI

The diagnosis of A-T can be confirmed by the absence or deficiency of ATM protein and/or ATM kinase activity in cultured cell lines established from lymphocytes or skin biopsies [138, 139] or the identification of pathological mutations in the *ATM* gene. These more specialized tests are not always needed, but are particularly helpful if an individual’s symptoms are atypical.

As whole exome sequencing becomes standard clinical practice for individuals with unusual and/or unexplained symptoms, it is likely that more people with mild forms of A-T will be diagnosed ([140] and unpublished observations). This will necessarily change our views about the phenotypic expression of A-T.

### Differential diagnosis

There are several other disorders with similar symptoms or laboratory features that physicians may consider when diagnosing A-T [2]. The three most common disorders that are sometimes confused with A-T are: cerebral palsy, congenital ocular motor apraxia and Friedreich’s ataxia. Each of these can be distinguished from A-T by the neurologic exam and clinical history (unpublished observations).

#### Cerebral palsy (CP)

CP describes any non-progressive disorder of motor function stemming from malformation or early damage to the brain [141]. Because most children suffering from A-T have stable neurologic symptoms for the first 4–5 years of life, a misdiagnosis of cerebral palsy is not uncommon [10]. However, milestones that have been accomplished and neurologic functions that have developed do not deteriorate in CP as they often do in children with A-T in the late pre-school years. In addition, most children with CP manifest regional or diffuse spasticity in a pattern not seen in A-T.

Those rare individuals that manifest a static disorder characterized by predominantly cerebellar features have been labeled as having “ataxic CP” (a term of uncertain nosology). Most individuals in this group do not begin walking at a normal age; however most children with A-T do, although they often “wobble” from the start. Children with ataxia caused by CP will not manifest the laboratory abnormalities associated with A-T.

#### Congenital ocular motor apraxia

Congenital ocular motor apraxia (COMA; Cogan OMA) is a rare disorder of delayed development of visual saccades [142]. COMA arises early and improves with time, whereas in A-T similar saccadic difficulties worsen over time, typically in early school years.

#### Friedreich’s Ataxia (FA or FRDA)

FRDA is the most common genetic cause of ataxia in children and the most prevalent autosomal recessive cerebellar ataxia [2]. In FRDA, ataxia typically appears between 10 and 15 years of age, and differs from A-T by the absence of telangiectasia and oculomotor apraxia, the early absence of tendon reflexes, a normal AFP, the frequent presence of scoliosis, and abnormal features on the EKG. FRDA and A-T also differ with regards to proprioception. Individuals with FRDA manifest difficulty standing in one place that is much enhanced by closure of the eyes (positive Romberg sign). This is not characteristic of A-T, even though those with A-T may have greater difficulty standing in one place with their eyes open ([10] and unpublished observations).

There are also other rare disorders that can be confused with A-T, either because of similar clinical features, a similarity of some laboratory features, or both. These include: ataxia oculomotor apraxia type 1 (AOA1), ataxia oculomotor apraxia type 2 (AOA2, also known as SCAR1), ataxia telangiectasia like disorder (ATLD) and Nijmegen breakage syndrome (NBS). A comparison of the clinical and laboratory features of these disorders can be found in Table 8.

**Table 8:** Clinical and Laboratory Features Of Rare Genetic Disorders that can be Confused With A-T (reviewed in [210] and [14])

	A-T	AOA1	AOA2	ATLD	NBS
Human gene	*ATM*	*APTX*	*SETX*	*MRE11*	*NBS1*
Radiosensitivity (type of DNA damage)	Yes (DSB)	Yes (SSB)	No (SSB?)	Yes (DSB)	Yes (DSB)
Immune deficiency	Yes	No	No	Mild [211]	Yes
Neurodegeneration	Yes	Yes	Yes	Yes	No
Early Neurodevelopment	Usually Normal	Normal	Normal	Normal	Microcephaly & Cognitive Impairment
Cancer risk	Yes	No	No	Unknown	Yes
Albumin	Normal	Low	Normal	Normal	Normal
AFP	High	Normal	High	Normal	Normal

*DSB* double strand break, *SSB* single strand break

Differentiation of these disorders is often possible with clinical features and selected laboratory tests. In cases where the distinction is unclear, DNA sequencing and/or protein assays (e.g. western blots or kinase assays to detect abnormal protein levels or activity) can be used to help make a definitive diagnosis.

### Pre-implantation genetic diagnosis, antenatal diagnosis and carrier identification

Pre-implantation genetic diagnosis (PGD) can avoid the birth of an affected child. PGD has been performed successfully for parents who have an affected child (or children) with A-T, and at least two case reports appear in the literature [143, 144].

Antenatal diagnosis and carrier detection can be cost effectively performed in families if the *ATM* mutations in an affected child have been identified. Antenatal diagnosis can also be performed using haplotype analysis if an unambiguous diagnosis has been made for the affected child. In this case, DNA polymorphisms within and around the *ATM* gene can be utilized even if the pathogenic mutations are not known.

Carrier testing in the general population, i.e. attempting to identify disease causing mutations in the *ATM* gene of an unrelated individual (for example, the spouse of a known A-T carrier), presents significant challenges. The *ATM* gene is extremely large and often contains polymorphisms which do not affect protein function. Clinicians cannot always predict if a specific variant will or will not cause disease.

### Newborn screening for SCID can detect A-T

The newborn screening test for severe combined immunodeficiency (SCID) detects T cell receptor and B cell kappa-deleting recombination excision circles (TRECs and KRECs), characteristic of lymphocyte deficiency, from infant dried blood spots. Other disorders characterized by a deficiency or absence of T and B cells can also be detected using this test [145]. Infants with T cell lymphopenia and A-T have been diagnosed with the SCID newborn screening test in combination with exome sequencing [146]. Although there is currently no disease modifying therapy or cure for A-T, diagnosis in infanthood allows for early family education and genetic counseling (see below) as well as early and more aggressive supportive care.

### Genetic counseling

Genetic counseling can provide education for families regarding the feasibility and potential consequences of genetic testing for A-T in siblings and other family members. Genetic counseling can also help with the interpretation of test results.

### Management

The management and treatment of A-T is symptomatic and supportive. Because A-T is a complex disease not all patients suffer from the same constellation of symptoms and they may vary in terms of the rate of disease progression and the on-set of complications. A brief review of treatments used for the various manifestations of A-T, as well as potential therapies including antioxidant and mutation-targeted approaches has been performed [147].

#### Neurologic problems

There is no treatment known to slow or stop the progression of the neurologic deficits associated with A-T. Physical, occupational and speech therapies as well as exercise may help maintain function but will not slow the course of neurodegeneration. Therapeutic exercises should not be used to the point of fatigue and should not interfere with activities of daily life.

Certain anti-Parkinson and anti-epileptic drugs may be useful in the management of symptoms. Commonly prescribed drugs include trihexyphenidyl (Artane), amantadine [148], baclofen and BOTOX® injections. Less commonly prescribed drugs that also may be beneficial include clonazepam [149], gabapentin and pregabalin (Lyrica) (reviewed in [11]). Various pharmaceutical interventions, (e.g. Riluzole [150]), have shown improvement in other cerebellar disorders. However, to date, their efficacy and the features of motor impairment that would best be targeted in A-T are not known. All drugs should be prescribed by a neurologist familiar with the assessment and treatment of individuals with movement disorders.

#### Immune problems

All individuals with A-T should have at least one comprehensive immunologic evaluation that measures the number and type of lymphocytes in the blood (T-lymphocytes and B-lymphocytes), the levels of serum immunoglobulins (IgG, IgA, and IgM) and antibody responses to T-dependent (e.g., tetanus, Hemophilus influenzae b) and T-independent (23-valent pneumococcal polysaccharide) vaccines. For the most part, the pattern of immunodeficiency seen in an A-T patient early in life (by age five) will be the same pattern seen throughout the lifetime of that individual [28, 32]; therefore, tests of immune function need not be repeated unless that individual develops more problems with infection. If infections are occurring in the lung, it is also important to investigate the possibility of dysfunctional swallow with aspiration.

##### Antibody deficiency

Problems with immunity can sometimes be overcome by immunization. Vaccines against common bacterial respiratory pathogens such as Hemophilus influenzae, pneumococci and influenza viruses are commercially available and often help to boost antibody responses, even in individuals with low immunoglobulin levels. If the individual continues to have problems with infections, gamma globulin therapy (IV or subcutaneous infusions) may be of benefit. The need for additional immunizations (especially with pneumococcal and influenza vaccines), antibiotics to provide prophylaxis from infections, and/or gamma globulin therapy should be determined by an expert in the field of immunodeficiency or infectious diseases.

In people with A-T who have low levels of IgA, further testing should be performed to determine if the IgA level is low or completely absent. If IgA is absent, there is a slight, albeit debatable, increase in the risk for a transfusion reaction. “Medical Alert” bracelets are not necessary, but the family and primary physician should be aware that if there is an elective surgery requiring red cell transfusion, the cells should be washed to decrease the risk of an allergic reaction.

##### Gammopathy/elevated immunoglobulin levels

A small number of people with A-T develop an abnormality in which one or more types of immunoglobulin are increased far beyond the normal range. In a few cases, the immunoglobulin levels can be increased so much that it causes hyperviscosity [151]. Therapy for this problem must be tailored to the specific abnormality found and its severity.

##### Lymphopenia

Many people with A-T have low lymphocyte counts in the blood. This problem seems to be relatively stable with age, but seldom causes susceptibility to opportunistic infections. The one exception is that problems with chronic or recurrent warts and molluscum contagiosum are relatively common [28].

The number and function of T-lymphocytes should be re-evaluated if a person with A-T is treated with corticosteroid drugs such as prednisone for longer than a few weeks or is treated with chemotherapy for cancer. If lymphocyte counts are low in people taking those types of drugs, the use of prophylactic antibiotics is recommended to prevent opportunistic infections.

##### Normal antibody function and vaccination

If antibody function is normal, all routine childhood immunizations including live viral vaccines (measles, mumps, rubella and varicella) should be given. Recommended vaccines for individuals with A-T are listed in Table 9.

**Table 9:** Vaccine Recommendations for A-T

• If a person with A-T does not need gamma globulin replacement therapy, he/she should receive all standard childhood vaccines, including the live vaccines for measles, mumps, rubella and varicella-zoster viruses.
• The individual with A-T and all household members should receive the influenza (flu) vaccine every fall.
• People with A-T who are less than two years old should receive three doses of a pneumococcal conjugate vaccine (Prevnar) given at two month intervals.
• People older than two years who have not previously been immunized with Prevnar should receive two doses of Prevnar.
• At least 6 months after the last Prevnar has been given, and after the child is at least two years old, the 23-valent pneumococcal vaccine should be administered. Immunization with the 23-valent pneumococcal vaccine should be repeated approximately every five years after the first dose.

##### Cutaneous granulomas

Chronic cutaneous granulomas occur in less than 10% of people with A-T. These lesions have not been associated with an identifiable pathogen or other etiology [36], but can on occasion be painful, bleed, or erode down to muscle or bone. Treatments have included high potency topical corticosteroids and/or cyclosporine A for small superficial lesions. More extensive granulomas may respond to combination therapy (e.g. topical steroids plus IV gamma globulin therapy) [152], systemic inhibitors of tumor necrosis factor (TNF-alpha) [153] or direct injection of steroids into the site of the granulomatous lesions [154].

#### Pulmonary problems

Recognizing and treating causes of chronic lung disease can minimize morbidity and delay onset of respiratory symptoms (reviewed in [124]). To slow or prevent the development of chronic lung disease in A-T, early intervention for respiratory symptoms is recommended. Pulmonary function testing should be performed in all children starting at 6 years of age and continued on an annual basis. Although pulmonary function tests can be difficult to perform in this population due to bulbar weakness and delayed initiation of inspiratory breaths, studies have demonstrated that with adjustments to the technique, reproducible spirometry can be performed in most people with A-T [39, 40, 124].

In people with chronic or persistent respiratory symptoms unresponsive to therapy, consideration should be given to lung imaging to diagnose unsuspected bronchiectasis, fibrosis, interstitial lung disease and tumors of the chest. Low dose chest and sinus CT are currently available which can minimize exposure to ionizing radiation [124]. Alternatively, magnetic resonance imaging can be used in people with A-T to identify lung abnormalities [155]. However, use of MRI may require anesthesia in younger patients.

##### General considerations for infection management

Liberal use of antibiotics should be considered in people with A-T who have persistent upper and lower respiratory tract symptoms. As with cystic fibrosis, people with A-T who are colonized with or who intermittently grow bacteria from their respiratory secretions are more likely to develop bronchiectasis and have more frequent respiratory exacerbations triggered by respiratory viral illnesses.

Administration of antibiotics should be considered when children and adults have prolonged respiratory symptoms (greater than 7 days) following a respiratory illness, including those that begin with a viral illness. Antibiotic treatment also should be considered in children with chronic coughs that are productive of mucus, those who do not respond to aggressive pulmonary clearance techniques and in children with muco-purulent secretions from the sinuses or chest. Examination of respiratory secretions by induced sputum or bronchoscopy may direct antibiotic therapy to treat lower respiratory tract infections and prevent the development of bronchiectasis.

In individuals who have recurrent pneumonias, bronchiectasis, or low lung function the use of macrolides, inhaled aminoglycoside and/or fluoroquinolones can sometimes reduce exacerbations [156, 157] and slow chronic lung disease progression. People with A-T and ILD may be responsive to corticosteroids. In a retrospective study, ILD progression was attenuated with early use of systemic corticosteroids [42]. However, this has not been validated in a prospective study. Finally people with restrictive lung disease associated with A-T may also have a component of obstructive lung disease responsive to bronchodilators [158].

##### Clearance of oral and bronchial secretions

Clearance of bronchial secretions is essential for good pulmonary health and can help limit injury from acute and chronic lung infections [159]. For those individuals with A-T who have difficulty clearing oral and bronchial secretions, techniques that allow clearance of mucus can be helpful during respiratory illnesses; however, evaluation by a pulmonary specialist should first be performed to properly assess patient suitability.

Children and adults with increased bronchial secretions may benefit from routine chest therapy using the manual method, and a cappella device or a chest physiotherapy vest. Chest physiotherapy can help bring up mucus from the lower bronchial tree; however an adequate cough is needed to remove secretions. In people who have decreased lung reserve and a weak cough, use of an insufflator-exsufflator device may be useful as a maintenance therapy or during acute respiratory illnesses to help remove bronchial secretions from the upper airways.

##### Respiratory muscle strength

A small study of 11 individuals with A-T found that inspiratory muscle training could improve respiratory muscle strength and quality of life for people with A-T [160].

##### ERS international statement on the respiratory treatment of A-T

In November 2015, an international, multidisciplinary task force of the European Respiratory Society (ERS) published a “Statement on the multidisciplinary respiratory management of ataxia-telangiectasia” [38]. The statement reviews the published data on lung disease in A-T and makes recommendations for treatment.

#### Problems with anesthesia: peri- and post-operative risks

If possible, all people with A-T should have an anesthesia or pulmonary consult before undergoing any surgical procedure or study that requires anesthesia. In a small retrospective study of people with A-T who underwent anesthesia at a tertiary care center, few complications were noted. However, they found that 24% of patients required supplemental oxygen post anesthesia and that 44% had mild hypothermia [41]. People with a history of significant restrictive lung disease may require non-invasive ventilation (NIV) during the recovery period. When possible, all procedures requiring anesthesia should be performed at a tertiary care center that has surgical and anesthetic expertise in the care of individuals with chronic respiratory and neuromuscular disease.

#### Problems with feeding, swallowing and nutrition

Oral intake may be enhanced by teaching persons with A-T how to drink, chew and swallow more safely. Treatments for swallowing problems should be determined following evaluation by an expert in the field of speech-language pathology. Dieticians may help treat nutrition problems by recommending dietary modifications, including high calorie foods or food supplements. To decrease the duration of mealtimes, caregivers may need to prepare and present foods or liquids to facilitate self-feeding or feed the person with A-T. Liquids are often easier to drink from covered containers with straws versus from open cups. It may be easier to finger feed than use utensils.

A gastrostomy tube (G-tube or feeding tube) is recommended when any of the following occur: a child cannot eat enough to grow or a person of any age cannot eat enough to maintain weight; aspiration is problematic; mealtimes are stressful or too long, interfering with other activities [161].

Feeding tubes can decrease the risk of aspiration by enabling persons to avoid liquids or foods that are difficult to swallow. They also provide adequate calories without the stress and time commitment of prolonged meals. G-tubes do not prevent people from eating by mouth. People who undergo gastric tube placement should initially be re-fed very slowly to avoid aspiration due to gastroesophageal reflux. Once a tube is in place, the general goal should be to maintain weight at the 10-25th percentile.

It has been demonstrated that, when placed at an early age, G-tubes can be well tolerated. In addition, caregivers reported significant improvements in mealtime satisfaction and participation in daily activities following G-tube placement [161].

Two recent studies by specialized A-T clinical centers in Germany and Australia have demonstrated that the extent of nutritional compromise in A-T likely exceeds previous estimates. Both groups demonstrated that malnutrition, as measured by decreased body cell mass (BCM) is a significant problem in the majority of people with A-T ([162, 163]).

Together, these studies emphasize the very critical need for nutritional intervention in certain people with A-T, including early and ongoing nutritional support and education for families and caregivers.

#### Problems associated with the management of cancers

The special problems of managing cancer in the context of A-T are sufficiently complicated that treatment should be performed only in academic oncology centers and after consultation with physicians who have specific expertise in A-T. For example, standard cancer treatment regimens need to be modified to avoid the use of radiation therapy and radiomimetic drugs, as these are particularly cytotoxic for people with A-T [164]. The use of cyclophosphamide must be monitored very carefully because it has been associated with severe hemorrhage from telangiectasia that develop in the bladder ([147] and unpublished observations). Of note, even with treatment modifications, for some individuals with A-T and advanced stage cancers toxicity may still pose significant problems [165].

There are two reports in the literature of successful bone marrow transplants (BMTs) for the treatment of T-ALL and non-Hodgkin lymphoma in individuals with A-T [166, 167]; however, the use of BMT for the treatment of hematopoietic malignancies associated with A-T is an area of active discussion.

#### Eye and vision problems

Eye muscle surgery can correct the strabismus that is common in people with A-T and help improve quality of life. Drugs that may improve other eye abnormalities, such as 4-amino pyridine for nystagmus and vestibular deficits [168], have not been rigorously prescribed or tested in patients with A-T.

#### Orthopedic problems

Early treatment of foot deformities may slow their progression. Bracing or surgical correction sometimes improves stability at the ankle sufficient to enable an individual to walk with support, or bear weight during assisted standing transfers from one seat to another. Severe scoliosis is relatively uncommon, but probably does occur more often than in those without A-T. Spinal fusion is only rarely indicated.

#### Education and socialization

Most children with A-T have difficulty in school because of a delay in response time to visual, verbal or other cues, dysarthria, oculomotor apraxia and impaired fine motor control. Despite these problems, children with A-T often enjoy school if proper accommodations to their disability can be made. The decision about the need for special education classes or extra help in regular classes is highly influenced by the local resources available. Decisions about proper educational placement should be revisited as often as circumstances warrant. Despite their many neurologic impairments, most individuals with A-T are very socially aware and socially skilled, and thus benefit from sustained peer relationships developed at school. Some individuals are able to function quite well despite their disabilities and a few have graduated from college.

Many of the problems encountered will benefit from special attention, as problems are often related more to “input and output” issues than to intellectual impairment. Problems with eye movement control make it difficult for people with A-T to read, yet most fully understand the meaning and nuances of text that is read to them. Delays in speech initiation and lack of facial expression make it seem that they do not know the answers to questions. Reduction of the skilled effort needed to answer questions, and an increase of the time available to respond, is often rewarded by real accomplishment. It is important to recognize that intellectual disability is not regularly a part of the clinical picture of A-T although school performance may be suboptimal because of the many difficulties in reading, writing, and speech. Children with A-T are often very conscious of their appearance, and strive to appear normal to their peers and teachers.

Life within the ataxic body can be tiring. The enhanced effort needed to maintain appearances and increased energy expended in abnormal tone and extra movements all contribute to physical and mental fatigue. As a consequence, for some a shortened school day yields real benefits.

General recommendations for the education and socialization of children with A-T are outlined in Table 10.

**Table 10:** General Recommendations for the Education and Socialization of Children with A-T

• All children with A-T need special attention to the barriers they experience in school. In the United States, this takes the form of a formal IEP (Individualized Education Program).
• Children with A-T tend to be excellent problem solvers. Their involvement in how to best perform tasks should be encouraged.
• Speech-language pathologists may facilitate communication skills that enable persons with A-T to get their messages across (using key words vs. complete sentences) and teach strategies to decrease frustration associated with the increased time needed to respond to questions (e.g., holding up a hand) and inform others about the need to allow more time for responses. Traditional speech therapies that focus on the production of specific sounds and strengthening of the lip and tongue muscles ﻿are rarely helpful.
• Classroom aides may be appropriate, especially to help with scribing, transportation throughout the school, mealtimes and toileting. The impact of an aide on peer relationships should be monitored carefully.
• Physical therapy is useful to maintain strength and general cardiovascular health. Horseback therapy and exercises in a swimming pool are often well-tolerated and fun for people with A-T. However, no amount of practice will slow the cerebellar degeneration or improve neurologic function. Exercise to the point of exhaustion should be avoided.
• Hearing is normal throughout life. Books on tape may be a useful adjunct to traditional school materials.
• Early use of computers (preschool) with word completion software should be encouraged.
• Practicing coordination (e.g. balance beam or cursive writing exercises) is not helpful.
• Occupational therapy is helpful for managing daily living skills.
• Allow rest time, shortened days, reduced class schedule, reduced homework, modified tests as necessary.
• Like all children, those with A-T need to have goals to experience the satisfaction of making progress.
• Social interactions with peers are important, and should be taken into consideration for class placement. For everyone, long-term peer relationships can be one of the most rewarding parts of life; for those with A-T establishing these connections in school years can be helpful.

### Prognosis

Historically, individuals with A-T succumbed to their disease in childhood or the teenage years. However, the average life expectancy for individuals with A-T has improved, and continues to improve, with advances in care. In 2006, the average life expectancy was reported to be approximately 25 years [37]. The two most common causes of death are chronic lung disease (about one-third of cases) and cancer (about one-third of cases).

### Unresolved questions

#### General

Many unresolved questions exist with regards to the complexity and severity of A-T. For example, the effect of environmental factors, disease modifier genes, epigenetics, telomere length, and the gut microbiome on the presentation, severity and progression of the various manifestations of A-T remains unknown. In addition, each manifestation has its own unresolved questions and unmet needs. These are described in brief below.

#### Neurology and neurodegeneration

##### Developmental and degenerative deficits

Degenerative neurological deficits are those that manifest loss of previously established abilities over time. The concept of what comprises a “developmental defect” is somewhat more complex, and can refer to either: 1) those deficits that disrupt the process of development itself, or 2) those that are present and fixed early, but the nature of the deficit emerges as normal developmental processes unveil the already limited capacity. The neurologic problems associated with A-T may well represent a mixture of these different processes.

##### Neurodegeneration at the cellular level

It is not yet known why, despite the ubiquitous expression of ATM, certain neurons in the brain, such as cerebellar PCs, are so exquisitely sensitive to its loss while others appear not to be affected. The specific vulnerability, and relative health, of neurons despite the loss of ATM protein may be cell autonomous, due to differing intrinsic properties of the neurons themselves, or non-cell autonomous, i.e. related to interactions in a circuit or relationships of selected neurons to their supporting environment.

##### Neurodegeneration at the functional level

Within the brain as a whole, we do not understand the functional specificity of the neurodegeneration associated with A-T. For example: beyond the simple observation that anatomic changes are concentrated in the cerebellum, the circuits and extra-cerebellar brain regions involved in the neurodegenerative process are not known. The involvement of functions not traditionally ascribed to the cerebellum has long been observed, but whether this is itself true, or instead a function of the limited scope of information about the appearance of neurologic disease concentrated in the cerebellum, remains to be seen. Longitudinal neuroimaging studies, starting at a young age and early in the disease process and that track neuropathological changes over time, are also lacking.

##### Contribution of the periphery

The brain is supported and affected by other organs of the body. As with other neurodegenerative disorders, “peripheral” abnormalities, such as malnutrition, oxidative stress, inflammation, autoimmunity as well as aging and endocrine changes, may contribute to the neurodegenerative process. Optimal management of these other changes – many of which are amenable to treatment – may help minimize the neurologic manifestations.

#### Immunodeficiency

One of the most obvious unresolved questions with regards to patients with classic A-T is why some suffer from immunodeficiencies like hypogammopathies and lymphopenia while others do not.

Additionally, a recent study of the incidence of cancers in a national cohort of French patients with A-T found low levels of IgA in those patients who developed lymphoid cancers as compared to patients who developed carcinomas or those without cancers [48]. This observation raises the question as to whether low IgA levels are a risk factor or biomarker for the development of lymphoid malignancies in A-T.

Immunodeficiency, specifically low IgG and low IgA levels in combination with elevated IgM levels, may also be a risk factor for a worsening overall disease course [169].

#### Pulmonology

There exist many gaps in our knowledge regarding pulmonary disease in A-T. In general, there is a need for: clinical methods to identify those at increased risk for pulmonary decline and disease; optimal protocols for treatment of lung disease; alternative lung imaging techniques (MRI versus CT) for the routine monitoring of pulmonary disease; and the banking of lung tissue from patients with A-T and pulmonary disease. The contribution of inflammation to lung disease and the direct effect of ATM loss on lung epithelium are currently areas of active research. Unresolved questions related to recurrent sinopulmonary disease and bronchiectasis, ILD and bulbar weakness have been previously reviewed [124]. With particular regard to neuromuscular weakness, there is a need for cost/benefit assessment of exercises or regimens that strengthen the upper body including: postural intervention; weight lifting; respiratory therapy including inspiratory and expiratory muscle training; and Lee Silverman Voice Therapy.

#### Cancers

Cancer is not a uniform manifestation of A-T, therefore the identification of biomarkers and risk factors for the development of malignancies in A-T would be valuable. A study in Atm deficient mice demonstrated relationships between housing with sterile or non-sterile diet, water and bedding, the intestinal microbiome and the onset of lymphoma in these animals [170]. Since the intestinal microbiome has been shown to contribute to basal levels of inflammation and oxidative stress, this study adds to the growing body of evidence that malignancy can be influenced by such factors.

The development of less toxic treatment regimens for cancers that occur in the context of A-T is a critical need. Although these cancers can be successfully treated, consequences of therapy including late onset adverse events often occur. Standard guidelines for patient assessment before therapy and supportive care during and after therapy are also lacking. Although challenging, the development of standard protocols for the treatment of the most common type of cancers in A-T (e.g. diffuse large B cell lymphoma) would be valuable.

There is also a need for centralized pathology review and the routine genotyping and banking of tumor tissue from patients. A paucity in the latter has hampered our understanding of the biochemical pathways involved in the development of cancers in the context of A-T and consequently our ability to develop targeted therapies.

## Conclusions

Since its formal designation as a disease entity in 1957, a tremendous amount has been learned about the clinical manifestations of A-T, and advances in clinical care have significantly improved the average life span of individuals suffering from this disease.

### Clinical guidance document for A-T

In October 2014, a clinical guidance document on the diagnosis and treatment of ataxia telangiectasia in children was published by the UK A-T Society [88]. Written by specialists at the pediatric A-T Specialist Center in Nottingham, UK, the guidance is aimed at non-specialists to encourage a consistent multidisciplinary approach to treating A-T. In addition to the key clinical areas of genetics, neurology, pulmonary care, immunology and cancer, the document covers physical therapy, dietary management and the implications of surgery in people with A-T.

### International A-T patient registries

The field of A-T clinical research would benefit greatly from an international database for the storing and sharing of de-identified patient demographic information and clinical data. Ideally, such a database would house information related to the different facets of A-T including, but not limited to: immunology, genetics and genomic data, neurology and neuroimaging, pulmonology, cancer and growth and nutrition. Access to information on this scale would greatly improve our understanding of the natural history of A-T, facilitate retrospective as well prospective clinical studies, and readily allow clinicians to select appropriate cohorts of individuals for multi-site, therapeutic trials.

#### Global A-T family data platform

To address this need, and to make data about individuals with A-T rapidly accessible to scientists and physicians for analysis, the Global A-T Family Data Platform was launched in July of 2016 [171]. Parents or guardians of children with A-T, or individuals with A-T themselves, share their medical information and have the option to provide a sample of their or their child’s saliva for whole genome sequencing. With advice from a Scientific and Medical Advisory Board, a Family Oversight Committee - including family members from 10 different countries - oversees the mission and activities of the Platform.

#### International A-T registry

In parallel to the Platform, an international A-T patient registry is being developed. Funded by a European Commission Horizon 2020 grant and overseen by a scientific board of clinical experts and patient representatives, the registry will contain baseline and longitudinal data provided by clinicians and clinical centers who treat individuals with A-T. The dataset will include the fields noted above. The international A-T advocacy community is taking steps to enable the linkage of data between the two registries and potentially other databases holding information on people with A-T.

### “ATM syndrome”

A 2015 historical review of A-T proposed “ATM Syndrome” as a new designation for A-T [172]. It remains to be seen if this will become an active topic for discussion amongst the international A-T clinical and advocacy communities.

## Abbreviations

AFP: Alpha-fetoprotein
AOA: Ataxia oculomotor apraxia
APTX: Aprataxin
A-T: Ataxia telangiectasia
ATLD: Ataxia telangiectasia like disorder
ATM: Ataxia telangiectasia mutated
BCM: Body cell mass
BMI: Body mass index
BMT: Bone marrow transplant
CCAS: Cerebellar cognitive affective syndrome
Cho: Choline
CNS: Central nervous system
COMA: Congenital ocular motor apraxia
CP: Cerebral palsy
Cr: Creatine
CT: Computed tomography
DBS: Deep brain stimulation
DDR: DNA damage response
dMRI: Diffusion magnetic resonance imaging
DNA: Deoxyribose nucleic acid
DSB: Double strand break
FA/FRDA: Friedreich’s Ataxia
FVC: Forced vital capacity
HRR: Homologous recombination repair
IGF: Insulin-like growth factor
ILD: Interstitial lung disease
IMT: Inspiratory muscle training
ITP: Immune thrombocytopenia
IV: Intravenous
MRE: Meiotic recombination
MRI: Magnetic resonance imaging
MSC: Melanocyte stem cell
NAA: N-acetyl aspartate
NAC: N-acetyl cysteine
NBS: Nijmegen breakage syndrome
NHEJ: Non-homologous end-joining
PET: Positron emission tomography
PGD: Pre-implantation genetic diagnosis
PIKK: PI3 Kinase-like kinase
RAD: Radiation sensitive
ROS: Reactive oxygen species
SCAR: non-Friedreich spinocerebellar ataxia, autosomal recessive 1
SCID: Severe combined immunodeficiency
SETX: Senetaxin
SSB: Single strand break
TDP: Tyrosyl-DNA phosphodiesterase
TNF: Tumor Necrosis Factor
UV: Ultraviolet
VOR: Vestibulo-ocular reflex
